# Supplementary material for: Whole-exome sequencing in a Japanese multiplex family identifies new susceptibility genes for intracranial aneurysms
Source: PLoS One. 2022 Mar 17;17(3):e0265359. doi: 10.1371/journal.pone.0265359 (PMC8929693; doi:10.1371/journal.pone.0265359)
Supplement: S1 File — (DOCX) [file pone.0265359.s001.docx]

**Supporting Information**

Whole-exome sequencing in a Japanese multiplex family identifies new susceptibility genes for intracranial aneurysms

Tatsuya Maegawa, MD^1,2^; Hiroyuki Akagawa, MD^1^; Hideaki Onda, MD^2,3^;

Hidetoshi Kasuya, MD^2^

^1^ Tokyo Women’s Medical University Institute for Integrated Medical Sciences (TIIMS), Tokyo, Japan

^2^ Department of Neurosurgery, Department of Neurosurgery, Tokyo Women’s Medical University Medical Center East, Tokyo, Japan

^3^ Division of Neurosurgery, Kofu Neurosurgical Hospital, Kofu, Yamanashi, Japan

Correspondence should be addressed to Akagawa H. (akagawa.hiroyuki@twmu.ac.jp)

**Table S1:** Representative variants in F2054 confirmed by Sanger sequencing.

| hg19 position | refGene | Sequence change | dbSNP rs ID | Genotype | | | | | | | HGVD ver.1.42^1^ | gnomAD exome^2^ | SIFT^3^ | Polyphen2 HumDiv^4^ |
| --- | --- | --- | --- | --- | --- | --- | --- | --- | --- | --- | --- | --- | --- | --- |
|  |  |  |  | III-1 | III-2 | III-4 | III-5 | III-6 | III-7 | IV-1 |  |  |  |  |
| 1p36.32 2316473 | *MORN1* [NM_024848] | c.481C>T (p.R161W) | rs777188997 | C/T | C/T | C/T | C/T | C/C | C/C | C/C | 0.0089 | 0.0003 | Tolerated | Probably damaging |
| 3p22.2 39119698 | *WDR48* [NM_020839] | c.1043C>T (p.P348L) | rs747971922 | C/T | C/T | C/T | C/T | C/T | C/C | C/C | 0 | <0.0001 | Deleterious | Benign |
| 3q13.13 108147562 | *MYH15* [NM_014981] | c.3539A>C (p.E1180A) | rs141309745 | A/C | A/C | A/C | A/A | A/A | A/A | A/A | 0.0185 | 0.0002 | Deleterious | Probably damaging |
| 3q22.1 132438619 | *NPHP3* [NM_153240] | c.449C>T (p.A150V) | rs142663818 | C/T | C/T | C/T | C/T | C/T | C/T | C/T | 0.0232 | 0.0016 | Deleterious | Probably damaging |
| 4q13.3 74283866 | *ALB* [NM_000477] | c.1490T>A (p.V497D) | - | T/A | T/A | T/A | T/A | T/T | T/T | T/A | 0 | 0 | Deleterious | Probably damaging |
| 4q24 106888602 | *NPNT* [NM_001184692] | c.1515+1G>A (exon10 skipping) | rs776559543 | G/A | G/A | G/A | G/A | G/A | G/G | G/A | 0 | 0.0002 | - | - |
| 5p13.1 39377375 | *DAB2* [NM_001343] | c.1514C>T (p.T505I) | rs145194026 | C/T | C/T | C/T | C/T | C/C | C/C | C/T | 0.0174 | 0.0018 | Tolerated | Benign |
| 5q11.2 55479405 | *ANKRD55* [NM_024669] | c.115G>C (p.D39H) | rs201139565 | G/C | G/C | G/C | G/C | G/C | G/G | G/C | 0.0064 | 0.0008 | Tolerated | Probably damaging |
| 5q11.2 56778312 | *ACTBL2* [NM_001017992] | c.223G>A (p.G75R) | - | G/A | G/A | G/A | G/A | G/A | G/G | G/A | 0 | 0 | - | Probably damaging |
| 5q13.2 71494310 | *MAP1B* [NM_005909] | c.5128T>C (p.S1710P) | rs747229477 | T/C | T/C | T/C | T/C | T/C | T/T | T/C | 0.0019 | <0.0001 | Deleterious | Possibly damaging |
| 7q21.11 80290426 | *CD36* [NM_001001548] | c.329_330del (p.T111Sfs*22) | rs572295823 | del/- | -/- | del/- | del/- | del/- | -/- | -/- | 0.0096 | 0.0011 | - | - |
| 9q22.31 94538052 | *ROR2* [NM_004560] | c.146G>T (p.G49V) | rs201991252 | G/T | G/T | G/T | G/T | G/G | G/G | G/G | 0.0063 | 0.0001 | Deleterious | Possibly damaging |
| 12q13.13 54422555 | *HOXC6* [NM_004503] | c.250C>T (p.L84F) | rs767298048 | C/T | C/T | C/T | C/T | C/T | C/C | C/T | 0.0012 | <0.0001 | Tolerated | Probably damaging |
| 12q13.13 54422579 | *HOXC6* [NM_004503] | c.274T>A (p.L92I) | rs779640921 | T/A | T/A | T/A | T/A | T/A | T/T | T/A | 0.0012 | <0.0001 | Tolerated | Benign |
| 12q13.13 54894351 | *NCKAP1L* [NM_005337] | c.A248A>G (p.E83G) | - | A/G | A/G | A/G | A/G | A/G | A/A | A/G | 0 | 0 | Deleterious | Probably damaging |
| 12q24.32 126138151 | *TMEM132B* [NM_001366854] | c.2147C>T (p.S716L) | rs760140118 | C/T | C/T | C/T | C/T | C/T | C/C | C/T | 0.0033 | <0.0001 | Deleterious | Possibly damaging |
| 13q14.13 46287515 | *CBY2* [NM_001286342] | c.247C>A (p.P83T) | rs200515699 | C/A | C/A | C/A | C/A | C/A | C/C | C/A | 0.0018 | 0.0005 | Deleterious | Probably damaging |
| 13q22.2 76395464 | *LMO7* [NM_001306080] | c.2359C>T (p.P787S) | rs41286126 | C/T | C/T | C/T | C/T | C/C | C/C | C/T | 0.0606 | 0.063 | Tolerated | Benign |
| 13q22.2 76395486 | *LMO7* [NM_001306080] | c.2381A>G (p.D794G) | rs200351536 | A/G | A/G | A/G | A/G | A/A | A/A | A/G | 0.0179 | <0.0001 | Tolerated | Benign |
| Xp21.1 32509404 | *DMD* [NM_004006] | c.2612A>C (p.K871T) | rs398123899 | A/C | A/C | A/C | A/C | A/C | A/C | A/C | 0.0175 | 0.0001 | Tolerated | Benign |
| Xp11.23 49075345 | *CACNA1F* [NM_001256789] | c.2729T>C (p.L910P) | - | T/C | T/C | T/C | T/C | T/C | T/C | T/C | 0 | 0 | Deleterious | Probably damaging |

1: Allelic frequencies in the Human Genome Variation Database (https://www.hgvd.genome.med.kyoto-u.ac.jp/) release version 1.42^1^.

2: Allelic frequencies in the exome dataset of the Genome Aggregation Database (https://gnomad.broadinstitute.org/)^2^.

3, 4: SIFT and PolyPhen2 predictions were obtained from dbNSFP3.0a (https://sites.google.com/site/jpopgen/dbNSFP) using ANNOVAR (https://annovar.openbioinformatics.org/en/latest/)^3,4^.

**Tables S2:** Primer sequences used in the study.

**S2-1:** Primers used for the minigene assay of the *NPNT* c.1515+1G>A variant.

| Use | Primer title | Primer sequence (5' -> 3') |
| --- | --- | --- |
| pET01 insert | NPNT_ex10_Xho1_F | ATACTCGAGATTGGCACATACACACAGCC |
|  |  | *Xho*1 |
|  | NPNT_ex10_Spe1_R | TATACTAGTGGTGATCACAGGTTGCAATG |
|  |  | *Spe*1 |
| cDNA synthesis^1^ | cDNA primer 01 | GATCCACGATGC |
| Exon trap RT-PCR^2^ | PCR primer 02 (Forward) | GATGGATCCGCTTCCTGCCCC |
|  | PCR primer 03 (Reverse) | CTCCCGGGCCACCTCCAGTGCC |

1, 2: These primer sequences were determined using of the Exontrap system (MoBiTec GmbH), according to the manufacturer’s instructions. https://www.mobitec.com/media/datasheets/mobitecgmbh/Exontrap-Handbook.pdf

**S2-2:** Primers used for exon sequencing of *NPNT* (NM_001184692).

| Name | Forward primer (5' -> 3') | Reverse primer (5' -> 3') | Product size |
| --- | --- | --- | --- |
| NPNT_exon1 | ACCACCCCAACCTGTTCCT | GGGAAAAGTGACCCAAGACA | 205 |
| NPNT_exon2 | TTGTTGGGTGTGGTTGACA | GGTGAAGCCATGAACAAGGT | 272 |
| NPNT_exon3 | CTAGTGCACGACATCAATGC | CAGCAAAATGGCAGCTCA | 296 |
| NPNT_exon4 | TGGCTTACATTAGTGCTGAGG | AGCACAACAGAAACAAGCCC | 235 |
| NPNT_exon5 | TCCATTACAGATTTTGTTTTCTACC | TCCCTTTATCCTACAGAGGCTG | 228 |
| NPNT_exon6 | CCCACTCTGTCTTAACATACCC | CAGCCATCACCACAATTACG | 270 |
| NPNT_exon7 | TGTAGATCATCACATTGCCAAA | GCAATGGCCTTTGTGTAATCC | 300 |
| NPNT_exon8 | TGCTTCACTTTTTAGGGAGG | TGGCCTGTGGTTGAAAAGTC | 520 |
| NPNT_exon9 | GAACACTTGTTGTCTAGATACCCC | TTAGCAGCAGTGGAGTCTTCAG | 269 |
| NPNT_exon10 | CTGTCCATGCTGCTTGTTCT | GAAAGGAAAACGTCCCAGGT | 371 |
| NPNT_exon11 | CATTAGAAAGGGGCAGAGGA | CAGAAAAACCTGGGTCACCA | 296 |

**S2-3:** Primers used for gene expression analysis of *CBY2*.

| Name | Forward primer (5' -> 3') | Reverse primer (5' -> 3') | Product size |
| --- | --- | --- | --- |
| CBY2_isoform1 | TCCAATCAGTGTGGTTCTACCTC | AGCGGTTCAGTGGATAGGAGTG | 152 |
| CBY2_isoform2 | GGAGGAATCCAATGCAGAGA | TCTAAAGGCACGGAGGAGAA | 165 |
| GAPDH | GCACCGTCAAGGCTGAGAAC | ATGGTGGTGAAGACGCCAGT | 142 |

**S2-4:** Primers used to generate the CBY2 isoform2 (Q8NA61-2) vectors.

| Use | Primer title | Primer sequence (5' -> 3') |
| --- | --- | --- |
| pDsRed-  Monomer-C1 insert | CBY2_iso2_Nhe1_F | CGCTAGCATGCAACCAGAGGGGTTG |
|  |  | *Nhe*1 |
|  | CBY2_iso2_Age1_R | GACCGGTACCCTGCTAGGCTTCTTGG |
|  |  | *Age*1 |
| p.P83T mutagenesis | p.P83T_inverse_F | ACGCGGGTGCAGCTCAGCGACGAGATG |
|  | p.P83T_inverse_R | CGGGTTGTAGTCCAGCTCCAGGTCG |

**S2-5:** Primers used for exon sequencing of *CBY2* (NM_152719, NM_001286342).

| Name | Forward primer (5' -> 3') | Reverse primer (5' -> 3') | Product size |
| --- | --- | --- | --- |
| CBY2_cds1a | TAGTCACAGCACCCACAAGC | GCTGTACCTGCTTCCCTGTC | 237 |
| CBY2_cds2 | TTTTAGGCGAGCAATTGAGG | TTTGAAGGAGGAGATGGGG | 223 |
| CBY2_cds1b | TGCTGCTATGTCACAAAGGG | CCGCTGAACATACTTGCCTA | 225 |
| CBY2_cds3-1 | CAAGTGTGTCAGTCCCATCG | TGGAACACGAACATCTCGTC | 288 |
| CBY2_cds3-2 | TTCTCCTCCGTGCCTTTAGA | GTTCTCCTTGCTGAGCATCC | 297 |
| CBY2_cds3-3 | GCTGCAGGAGGAGAACAAGT | GCCTGCCTCATCTCTCTCAG | 594 |
| CBY2_cds3-4 | AGGAGGACTCCAAGGAGCTG | GGATTCTCTTTTGCCCAGTG | 457 |

**Table S3:** Association analysis of common variants in *CBY2*.

| Sequence change | db SNP rs ID | Case (501) | | | Control (323) | | | P value^*^ |
| --- | --- | --- | --- | --- | --- | --- | --- | --- |
|  |  |  |  |  |  |  |  |  |
| c.749C>A (p.Ser250Ter) | rs79707842 | CC | CA | AA | CC | CA | AA | 0.346 |
|  |  | 347 | 131 | 23 | 227 | 90 | 6 |  |
| c.877A>G (p.Lys293Glu) | rs7317245 | AA | GA | GG | AA | GA | GG | 0.838 |
|  |  | 89 | 246 | 166 | 59 | 152 | 112 |  |

* Allelic associations between SNPs and IA were evaluated using Fisher’s exact test.

We performed exact tests of the Hardy-Weinberg equilibrium using PLINK version 1.07 (http://zzz.bwh.harvard.edu/plink/index.shtml)^5^. The genotypic frequencies of these SNPs were considered to meet the Hardy-Weinberg expectations in both the patients and the controls (P>0.001)^6^.


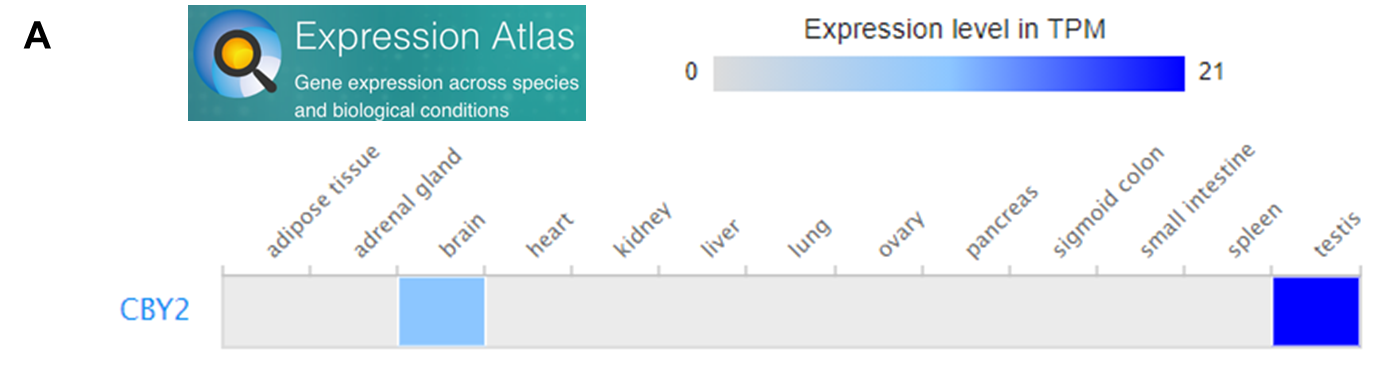


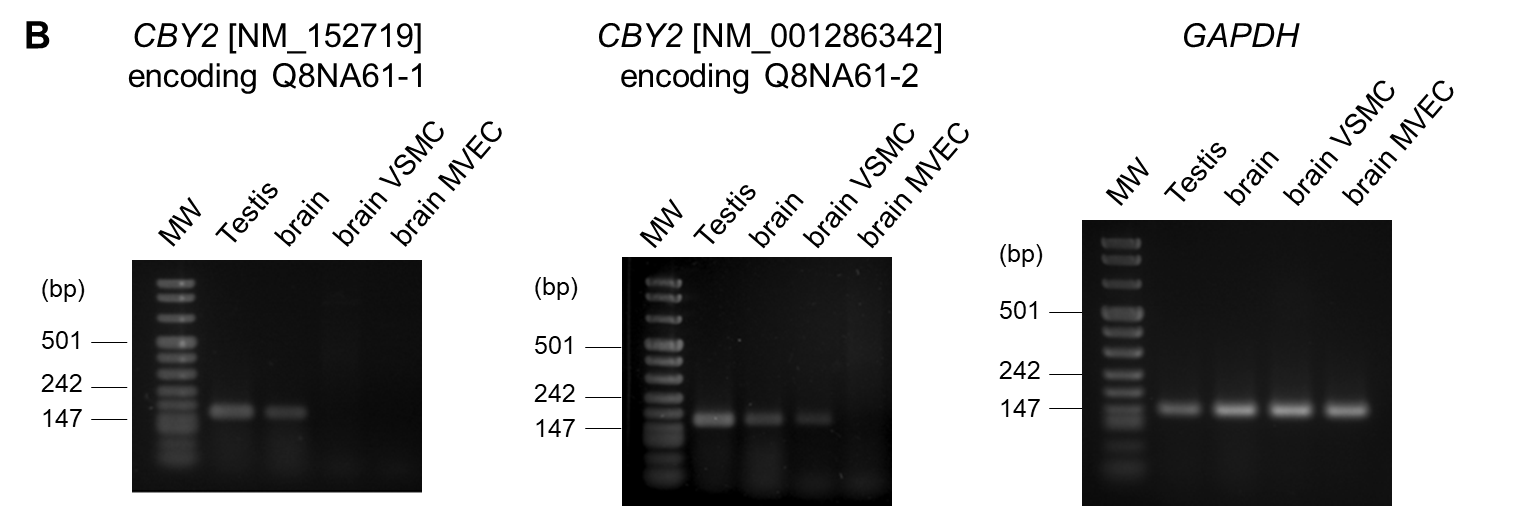


**Figure S1: *CBY2* expression in human tissues.** (A) *CBY2* is expressed in the testis and the brain according to the ENCODE data (Michael Snyder's lab) provided in the Expression Atlas (https://www.ebi.ac.uk/gxa/home)^7^. (B) RT-PCR products were visualized by 3% agarose gel electrophoresis. *CBY2* expression in human VSMC was transcription variant-specific (NM_001286342 encoding Q8NA61-2). Abbreviations: TPM, transcripts per million; MW, molecular weight marker VIII (Roche); VSMC, vascular smooth muscle cell; MVEC, microvascular endothelial cell, RT-PCR; reverse transcription-polymerase chain reaction.

**
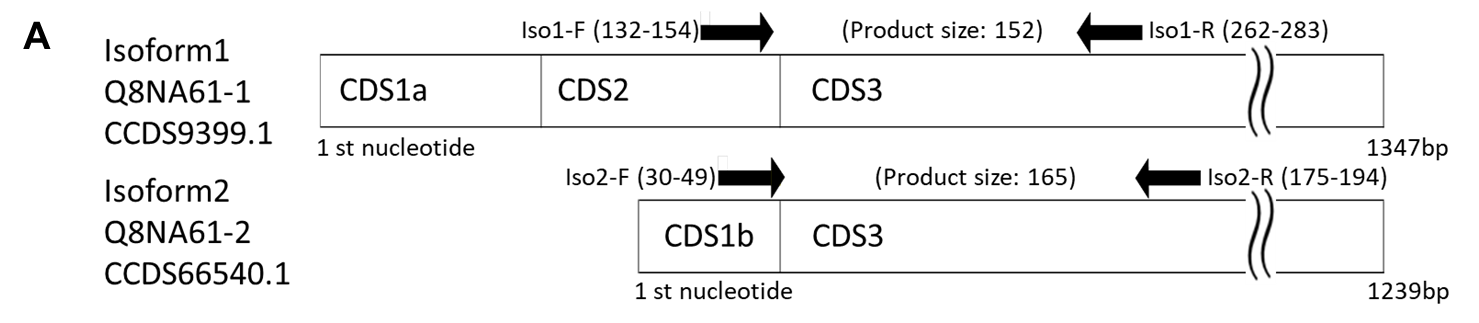
**


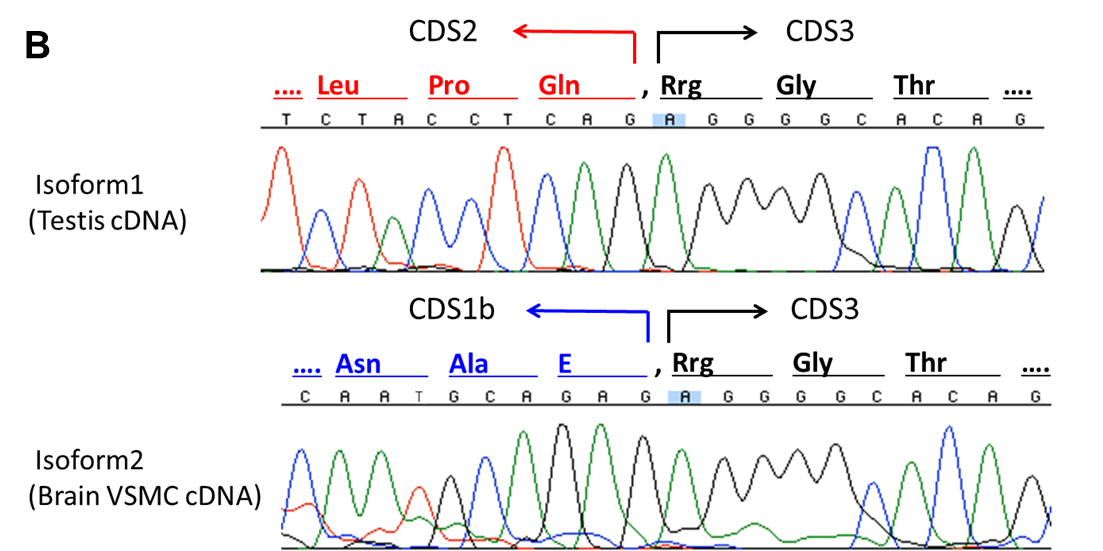


**Figure S2: Direct sequencing of isoform-specific RT-PCR products of *CBY2*.** (A) Alternative exon usage of *CBY2* isoforms. Arrows represent isoform-specific RT-PCR primers listed in Table S2-3. (B) The RT-PCR products represented in Figure S1-B were analyzed by Sanger sequencing. *CBY2* expression in the brain VSMCs was confirmed to be isoform2-specific. Abbreviations: CDS, coding sequence; VSMC, vascular smooth muscle cell.

**
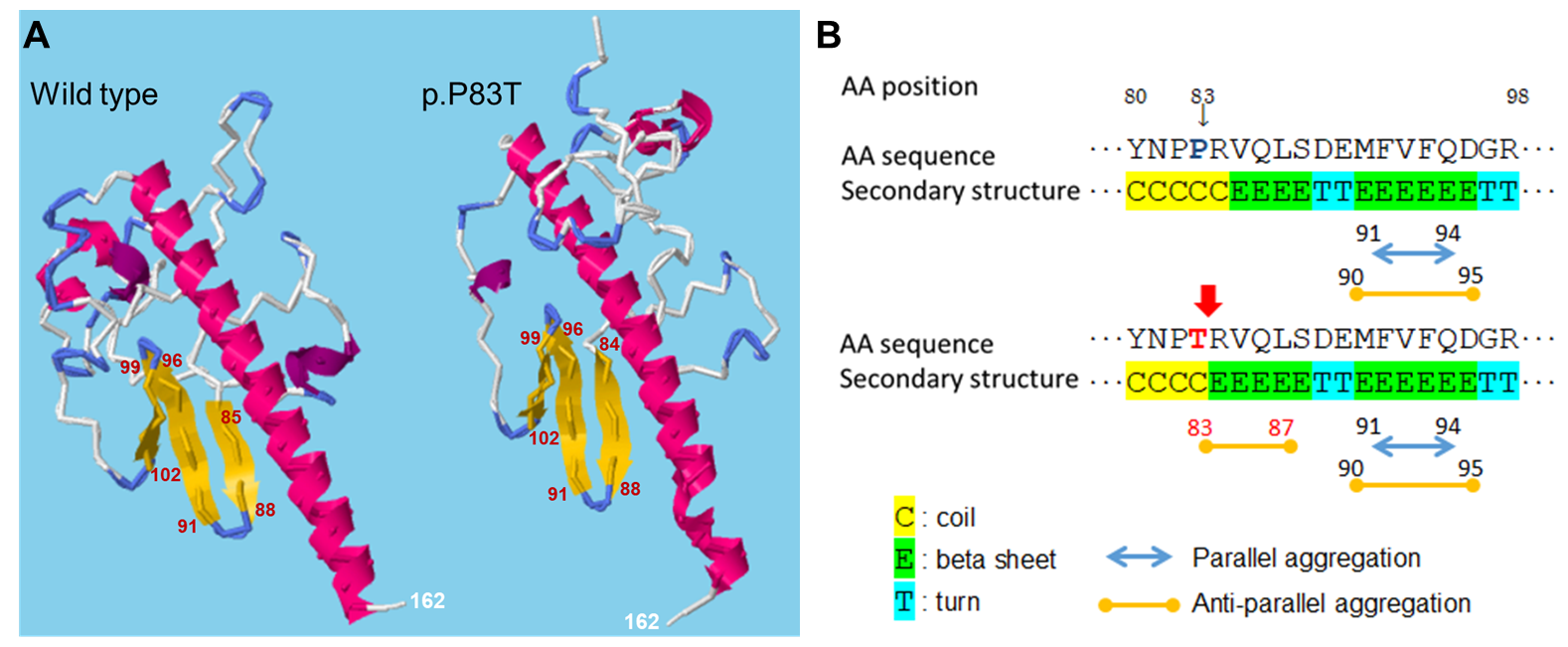
**

**C**

**
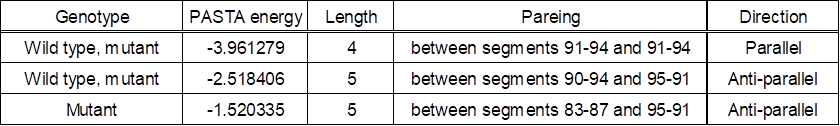
**

**Figure S3: Prediction of 3D protein structure and aggregation propensity.** (A, B) The p.Pro83Thr substitution in CBY2 elongates the neighboring stretches of beta strand according to the RaptorX web server (http://raptorx.uchicago.edu/)^8^. White, coil; blue, turn; magenta, alfa helix; yellow, beta sheet. (B, C) This elongated beta strand forms a cross-beta aggregation motif according to the Prediction of Amyloid STructure Aggregation 2.0 (PASTA 2.0, http://old.protein.bio.unipd.it/pasta2/)^9^. The amino acid sequence from the start codon to the end of the first coiled-coil domain (1-162) of CBY2 isoform2 (Q8NA61-2) was used for calculation. Abbreviations: AA, amino acid.

**References**

1. Higasa K, Miyake N, Yoshimura J, Okamura K, Niihori T, Saitsu H, Doi K, Shimizu M, Nakabayashi K, Aoki Y, Tsurusaki Y, Morishita S, Kawaguchi T, Migita O, Nakayama K, Nakashima M, Mitsui J, Narahara M, Hayashi K, Funayama R, Yamaguchi D, Ishiura H, Ko WY, Hata K, Nagashima T, Yamada R, Matsubara Y, Umezawa A, Tsuji S, Matsumoto N, Matsuda F. Human genetic variation database, a reference database of genetic variations in the Japanese population. J Hum Genet. 2016;61(6):547-53.
2. Karczewski KJ, Francioli LC, Tiao G, Cummings BB, Alföldi J, Wang Q, Collins RL, Laricchia KM, Ganna A, Birnbaum DP, Gauthier LD, Brand H, Solomonson M, Watts NA, Rhodes D, Singer-Berk M, England EM, Seaby EG, Kosmicki JA, Walters RK, Tashman K, Farjoun Y, Banks E, Poterba T, Wang A, Seed C, Whiffin N, Chong JX, Samocha KE, Pierce-Hoffman E, Zappala Z, O'Donnell-Luria AH, Minikel EV, Weisburd B, Lek M, Ware JS, Vittal C, Armean IM, Bergelson L, Cibulskis K, Connolly KM, Covarrubias M, Donnelly S, Ferriera S, Gabriel S, Gentry J, Gupta N, Jeandet T, Kaplan D, Llanwarne C, Munshi R, Novod S, Petrillo N, Roazen D, Ruano-Rubio V, Saltzman A, Schleicher M, Soto J, Tibbetts K, Tolonen C, Wade G, Talkowski ME; Genome Aggregation Database Consortium, Neale BM, Daly MJ, MacArthur DG. The mutational constraint spectrum quantified from variation in 141,456 humans. Nature. 2020;581(7809):434-43.
3. Liu X, Wu C, Li C, Boerwinkle E. dbNSFP v3.0: A One-Stop Database of Functional Predictions and Annotations for Human Nonsynonymous and Splice-Site SNVs. Hum Mutat. 2016;37(3):235-41.
4. Wang K, Li M, Hakonarson H. ANNOVAR: functional annotation of genetic variants from high-throughput sequencing data. Nucleic Acids Res. 2010;38(16):e164.
5. Purcell S, Neale B, Todd-Brown K, Thomas L, Ferreira MA, Bender D, Maller J, Sklar P, de Bakker PI, Daly MJ, Sham PC. PLINK: a tool set for whole-genome association and population-based linkage analyses. Am J Hum Genet. 2007;81(3):559-75.
6. Wigginton JE, Cutler DJ, Abecasis GR. A note on exact tests of Hardy-Weinberg equilibrium. Am J Hum Genet. 2005;76(5):887-93.
7. Papatheodorou I, Moreno P, Manning J, Fuentes AM, George N, Fexova S, Fonseca NA, Füllgrabe A, Green M, Huang N, Huerta L, Iqbal H, Jianu M, Mohammed S, Zhao L, Jarnuczak AF, Jupp S, Marioni J, Meyer K, Petryszak R, Prada Medina CA, Talavera-López C, Teichmann S, Vizcaino JA, Brazma A. Expression Atlas update: from tissues to single cells. Nucleic Acids Res. 2020 Jan 8;48(D1):D77-D83.
8. Källberg M, Wang H, Wang S, Peng J, Wang Z, Lu H, Xu J. Template-based protein structure modeling using the RaptorX web server. Nat Protoc. 2012 Jul 19;7(8):1511-22.
9. Walsh I, Seno F, Tosatto SC, Trovato A. PASTA 2.0: an improved server for protein aggregation prediction. Nucleic Acids Res. 2014;42(Web Server issue):W301-7.
