# Supplementary material for: Whole-exome sequencing in a Japanese multiplex family identifies new susceptibility genes for intracranial aneurysms
Source: PLoS One. 2022 Mar 17;17(3):e0265359. doi: 10.1371/journal.pone.0265359 (PMC8929693; doi:10.1371/journal.pone.0265359)
Supplement: S1 Raw images — (PDF) [file pone.0265359.s002.pdf]

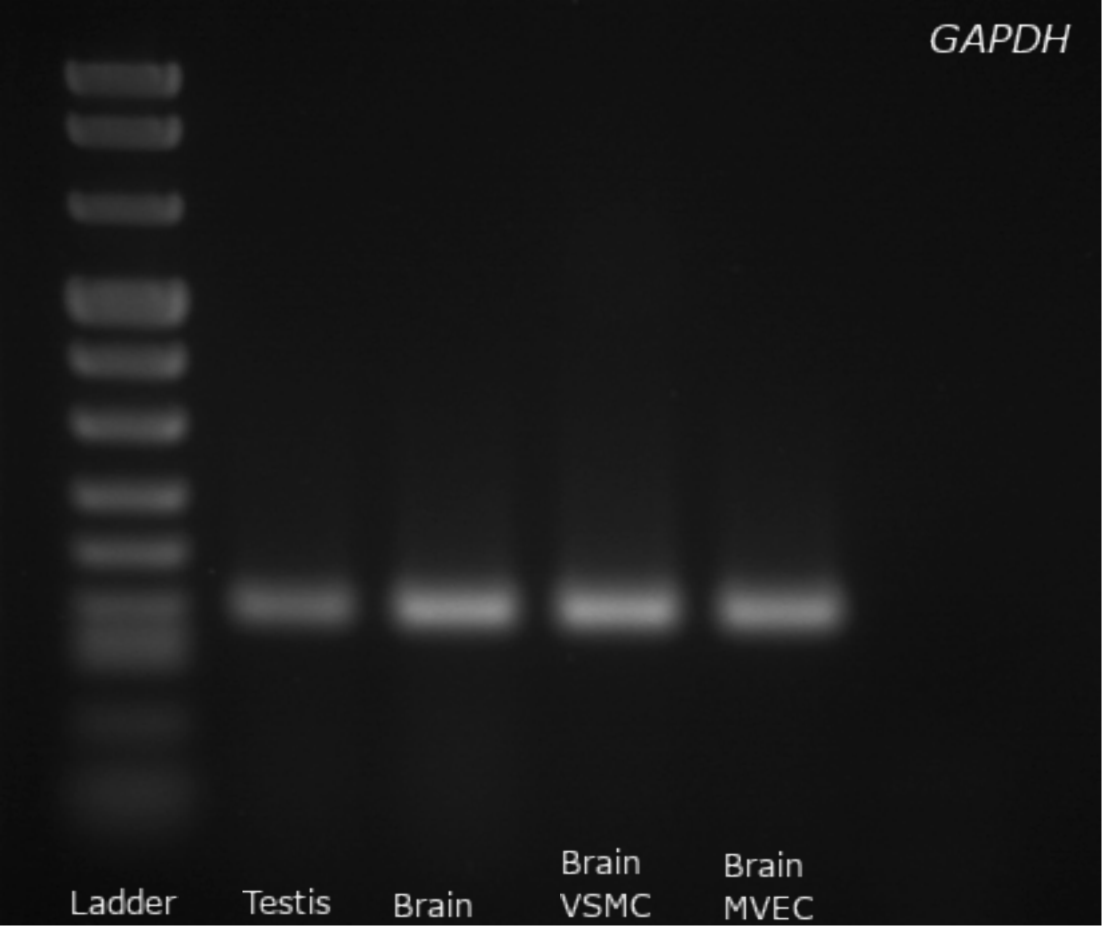
GAPDH
Ladder
Testis
Brain
Brain VSMC
Brain MVEC

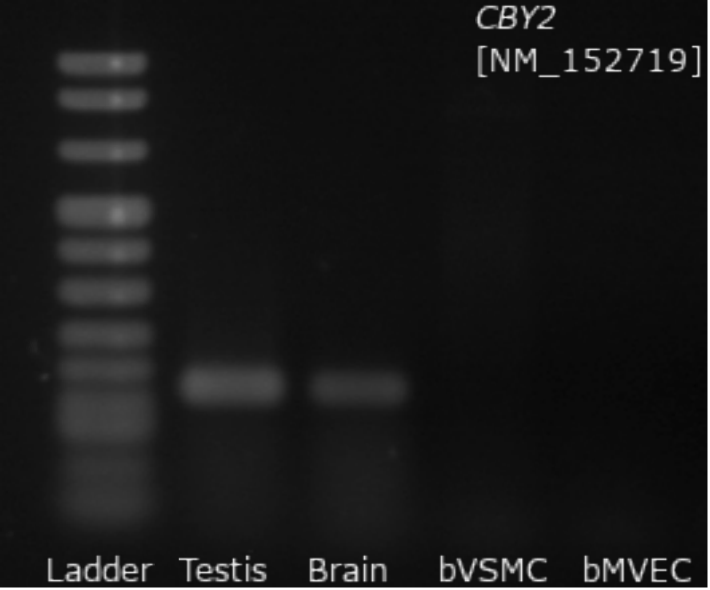
CBY2
[NM_152719]
Ladder
Testis
Brain
bVSMC
bMVEC

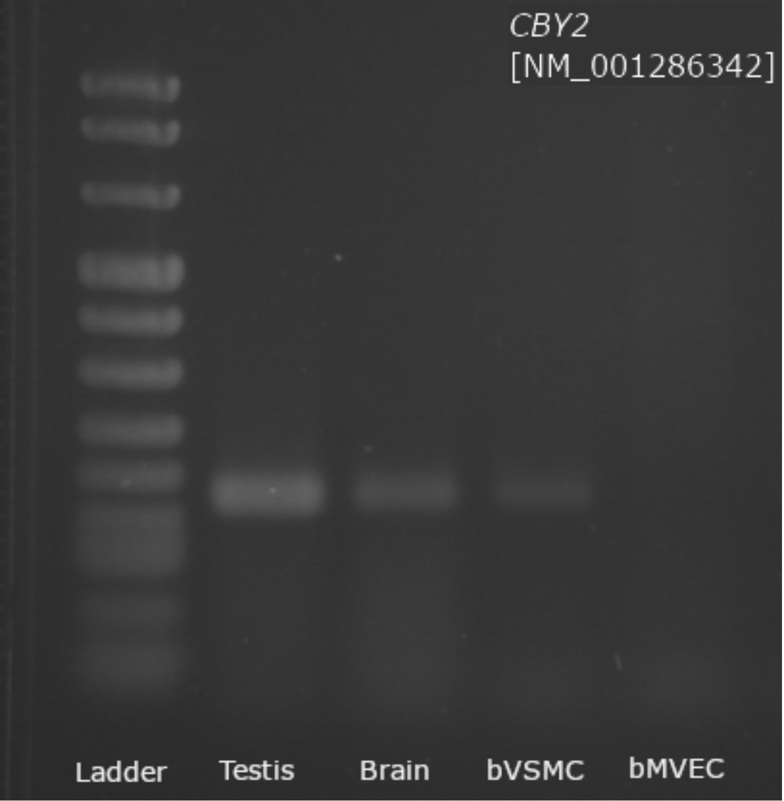
CBY2
[NM_001286342]
Ladder
Testis
Brain
bVSMC
bMVEC
