# Supplementary material for: Whole-exome sequencing in a Japanese multiplex family identifies new susceptibility genes for intracranial aneurysms
Source: PLoS One. 2022 Mar 17;17(3):e0265359. doi: 10.1371/journal.pone.0265359 (PMC8929693; doi:10.1371/journal.pone.0265359)
Supplement: Attachment — Submitted filename: Response_to_Reviewers.docx [file pone.0265359.s003.docx]

**RESPONSE TO REVIEWER #1:**

We wish to express our appreciation to the Reviewer for his/her insightful comments, which have helped us to considerably improve our manuscript.

**Reviewer #1**: *This study brings forth causal variants in two genes NPNT and CBY2 for intracranial aneurysms. The authors have examined families and singletons and conducted expression studies for these genes. The focus has been on CBY2, within the locus identified in a different population and highly expressed in testis. I have the following comments about this manuscript:*

**Comment 1**: *Did the authors consider separate population frequency cut offs for predicted AD AR inheritance for each?*

**Response:** Thank you for this comment. We have added the following text (revised text lines 254-257):

“There were no homozygous or compound heterozygous candidates shared by the affected sisters (III-1, III-4, and III-5) with allelic frequencies less than 3% in the gnomAD and the Human Genome Variation Database (HGVD, https://www.hgvd.genome.med.kyoto-u.ac.jp/)”

**Comment 2**: *What population frequency database were referred to? I would include gnomAD, KAVIAR and any other population specific public database if available.*

**Response:** As we responded in comment 1 and presented in the Table S1, the Human Genetic Variation Database (HGVD, https://www.hgvd.genome.med.kyoto-u.ac.jp/) in the Japanese general population was referred to in addition to the gnomAD.

**Comment 3**: *In table 1, include population frequency for the alternate allele from gnomAD/ALFA like in Suppl table S1 etc.*

**Response:** Based on the reviewer’s comment, we have added the HGVD and gnomAD data in Table 1.

**Comment 4**: *The authors discuss the possibility of a complex pattern of inheritance including reduced penetrance for the multiplex families. I suggest that the authors include an inheritance pattern (autosomal dominant, recessive etc) explicitly in the Discussion to make their case stronger.*

**Response:** Based on the reviewer’s comment, we have changed the following text from (revised text lines 408-411):

“The accumulated genetic data from many other large pedigrees with IA will facilitate our understanding of the polygenic architecture of IA susceptibility.”

to

“Accumulated genetic data from many other large pedigrees with IA will facilitate our understanding of the polygenic architecture of IA susceptibility, which vastly differs from Mendelian inheritance such as autosomal dominant and recessive patterns.”

**Comment 5**: *Can the authors also include a hypothesis about the functional effect of the variants? In supplementary information, the data for splice site variant is shown for NPNT. It would be good to know if the CBY2 and NPNT variants are acting as loss or function or gain of function mutation in IA.*

**Response:** Based on the reviewer’s comment, we have added the following text (revised text lines 361-363) and reference to describe the presumed effect of the *CBY2* variant :

“Protein aggregation is involved in the etiology of various human diseases due to the loss of protein function, cytotoxicity, or acquisition novel aggregation-specific functions [44]. ”

“44. De Baets G, Van Doorn L, Rousseau F, Schymkowitz J. Increased aggregation is more frequently associated with human disease-associated mutations in neutral polymorphisms. PLoS Comput Biol. 2015;11(9):e1004374.”

The *NPNT* variants identified in our IA cases were loss-of-function, as mentioned in the discussion (revised text lines 353-355).

**Comment 6**: *The authors mention 'gene product' on many occasions throughout the manuscript. By that, do they mean mRNA or protein? If the latter, then it needs to be modified in the manuscript.*

**Response:** We strictly adhered to the guidelines for human gene nomenclature established by the HUGO Gene Nomenclature Committee (HGNC) throughout the manuscript, which is also recommended in the submission guidelines of *PLOS ONE* (https://journals.plos.org/plosone/s/submission-guidelines). The HGNC endorses the use of italics to denote genes, alleles and RNAs to distinguish them from proteins, and recommends that “protein and gene symbols should use the same abbreviation”. They further suggested that proteins should be referenced using non-italicized gene symbols to distinguish them from genes. Please see the following paper from HGNC.

Bruford EA, Braschi B, Denny P, Jones TEM, Seal RL, Tweedie S. Guidelines for human gene nomenclature. Nat Genet. 2020 Aug;52(8):754-758. doi: 10.1038/s41588-020-0669-3. PMID: 32747822; PMCID: PMC7494048.

**Comment 7**: *Correct syntax errors: Ex: Remove g for DNA sample of sister in line 267; Remove gDNA in line 386 etc.*

**Response:** We sincerely apologize for these errors. They have been corrected in the revised manuscript.

**Comment 8**: *I would suggest moving Figure S1 to the main manuscript.*

**Response:** Based on the reviewer’s comment, we moved Figure S1 to the main manuscript as Figure 2.

We wish to thank the Reviewer again for the valuable comments.

**RESPONSE TO REVIEWER #2:**

We wish to express our appreciation to the reviewer for his/her insightful comments, which have helped us to considerably improve our manuscript.

**Reviewer #2**: *I genuinely appreciate the effortful and highly systematic methodologies conducted in revealing novel susceptibility genes for intracranial aneurysms in the Japanese family. I do encourage the authors to conduct this well-elaborated methodology at a higher scale with increasing sample sizes and incorporating other non-Japanese candidates as possible in the near future.*

**Response:** We thank the reviewer for this important suggestion regarding our future research plan.
